# A Novel Method for Bearing Fault Diagnosis under Variable Speed Based on Envelope Spectrum Fault Characteristic Frequency Band Identification

**DOI:** 10.3390/s23094338

**Published:** 2023-04-27

**Authors:** Di Pei, Jianhai Yue, Jing Jiao

**Affiliations:** 1 School of Mechanical, Electronic and Control Engineering, Beijing Jiaotong University, Beijing 100044, China; pei_di@bjtu.edu.cn; 2 Locomotive & Car Research Institute, China Academy of Railway Sciences Corp. Ltd., Beijing 100081, China; jingjiao@bjtu.edu.cn

**Keywords:** envelope spectrum analysis, fault characteristic frequency band, variable speed, bearing fault diagnosis, vibration

## Abstract

Rolling element bearing (REB) vibration signals under variable speed (VS) have non-stationary characteristics. Order tracking (OT) and time-frequency analysis (TFA) are two widely used methods for REB fault diagnosis under VS. However, the effect of OT methods is affected by resampling errors and close-order harmonic interference, while the accuracy of TFA methods is mainly limited by time-frequency resolution and ridge extraction algorithms. To address this issue, a novel method based on envelope spectrum fault characteristic frequency band identification (FCFBI) is proposed. Firstly, the characteristics of the bearing fault vibration signal’s envelope spectrum under VS are analyzed in detail and the fault characteristic frequency band (FCFB) is introduced as a new and effective representation of faults. Then, fault templates based on FCFB are constructed as reference for fault identification. Finally, based on the calculation of the correlation coefficients between the envelope spectrum and fault templates in the extended FCFB, the bearing fault can be diagnosed automatically according to the preset correlation coefficient criterion. Two bearing VS experiments indicate that the proposed method can achieve satisfactory diagnostic accuracy. The comparison of OT and TFA methods further demonstrates the comprehensive superiority of the proposed method in the overall consideration of accuracy, diagnostic time, tachometer dependency, and automatic degree.

## 1. Introduction

Vibration signal analysis is widely used in rolling element bearing (REB) fault diagnosis [1]. When there is a local defect in a bearing, its vibration signal exhibits the characteristic of amplitude modulation, where the natural vibration of the mechanical system regarded as the carrier wave is modulated by the instantaneous impulse generated by the defect. The repetition frequency of the instantaneous impulse is called the bearing fault characteristic frequency (FCF). When the bearing geometry, defect location, and rotation speed are determined, the FCF is a constant value. Therefore, at constant speed, envelope demodulation combined with Fourier transform (FT), commonly called envelope spectrum analysis, is commonly used for bearing fault detection. Many advanced vibration signal preprocessing methods, such as spectral kurtosis and its improvement [2,3], empirical mode decomposition (EMD) as well as its modified algorithms [4,5,6,7], and deconvolution methods [8,9,10], have achieved good diagnostic results in combination with envelope spectrum analysis.

However, bearings do not always work at constant speed. Due to the change in the rotation speed, the FCF is not constant, which will cause smearing on the spectral line [11]. Therefore, it is difficult for fault diagnosis by observing the spectral lines located at FCF and its harmonics in the envelope spectrum similar to a constant speed condition. In order to carry out bearing fault diagnosis under VS, a large number of methods have been studied. Among them, as the most effective methods, order tracking and time-frequency analysis have been deeply studied and widely applied.

The computed order tracking (COT) proposed by K. R. Fyfe and E. D. S. Munck [12] overcomes the shortcomings of hardware OT and is the most popular OT method. COT resamples the time domain vibration signal at equal angular intervals to obtain the angular domain signal. Due to the quasi-stationary nature of the vibration signal in the angular domain, envelope spectrum analysis can be performed on the resampled angular signal, and fault diagnosis can be achieved by identifying the fault characteristic order (FCO) in the order envelope spectrum. Many COT-based studies were combined with vibration signal preprocessing and denoising methods, such as iterative envelope and low-pass filtering [13], spectral kurtosis [14,15], re-scaled stochastic resonance [16], and reverse sequence squared envelope spectrum [17], for bearing fault diagnosis under VS. Some scholars conducted research on the accuracy of the COT algorithm. Li et al. [18] believed that long data series would aggravate the cumulative periodic disturbance and affect the accuracy of COT. Cheng et al. [19] suggested that resampling will lead to the deformation of the angular domain vibration envelope, which will further lead to errors between the theoretical and actual FCO in envelope order spectrum.

Time-frequency analysis methods, such as short-time Fourier transform (STFT) and wavelet transform (WT), can reveal both time and frequency information on a 2D time-frequency plane, known as time-frequency representation (TFR) [20,21]. For the bearing fault vibration signal under VS, the instantaneous shaft rotational frequency (ISRF), instantaneous fault characteristic frequency (IFCF), and their harmonics manifest as time-frequency ridges in TFR. Therefore, fault diagnosis can be performed by identifying the time-frequency curves related to ISRF and IFCF in TFR without additional speed measurement and resampling processes. Huang et al. [22,23,24] proposed the multiple time-frequency curve extraction (MTFCE) method, of which the main idea is to extract ISRF and IFCF curves from the TFR of the vibration signal. Then, the average point-to-point ratios are calculated between the IFCF and ISRF curves and the ratios are compared with the theoretical fault characteristic coefficient (FCC) to determine the bearing condition. The time-frequency ridge extraction in [22,23,24] adopted the fast path optimization algorithm. Similarly, Wang et al. [25] and Tang et al. [26] used the respective cost function ridge detection algorithm and the local peak search method to obtain ridges from TFR. The TFA method with good energy concentration and less susceptibility to noise interference is the prerequisite for the accurate extraction of time-varying ISRF and IFCF curves. Hou et al. [27] proposed the sparse TFR method to suppress interference components in TFR. Feng et al. [28] treated the instantaneous amplitude and frequency of the vibration envelope as the signal to be analyzed and obtained the corresponding TFR through the concentration of the frequency and time method. Liu et al. [29] proposed the synchrosqueezing extraction transform algorithm, which increases the time-frequency energy concentration of TFR. In order to avoid the difficulty of extracting ISRF from TFR, Wang et al. [30] proposed the fault characteristic order spectrum analysis method based on FCO resampling via IFCF extracted from TFR. Meanwhile, TFA can be used to estimate the rotation speed from the vibration signal, which can be combined with COT to realize the tacholess OT [31,32,33]. In addition to OT and TFA methods, statistical time series methods are also widely employed for nonstationary signal processing; readers can refer to [34,35].

Although COT- and TFA-based methods have been widely used in bearing fault diagnosis under VS, they inevitably have some shortcomings. For COT-based methods, the rotation speed signal is necessary when calculating the resampling time. The additional speed measurement device increases the cost and complexity of the mechanical system. Secondly, the interpolation algorithm used in the resampling process will introduce an amplitude error. Last and most important, COT spectrum-based analysis suffers from close-order harmonic interference. More specifically, for some types of bearing, if different harmonic orders of different faults are too close (e.g., *mFCC*_1_ ≈ *nFCC*_2_, where *FCC* is the fault characteristic coefficient, *m* and *n* are harmonics) to distinguish from spectral lines, the determination of the specific fault type of bearing is difficult and even leads to incorrect results. In spite of the fact that the TFA-based methods do not require tachometers and resampling, the accuracy of the extracted instantaneous frequency is greatly affected by noise, time-frequency resolution, and ridge extraction algorithms. The combination of more advanced denoising, TFA, and ridge extraction algorithms will increase the complexity of the fault diagnosis method.

In view of the above, aimed at the aforementioned shortcomings existing in the OT- and TFA-based methods, this paper proposes a novel fault diagnosis method under VS by identifying the time-varying fault characteristic frequency band from the bearing vibration signal envelope spectrum. The proposed method is called the fault characteristic frequency band identification (FCFBI). FCFBI is inspired by the well-known envelope spectrum analysis for bearing fault diagnosis at constant speed. However, the current research on the envelope spectrum of the bearing vibration signal under VS has the following limitations:

1. There are few in-depth studies on the characteristics of the bearing vibration signal envelope spectrum under VS;
2. Many authors have reported that the variation of FCF will lead to spectrum smearing under VS [1,11,13,14,15,18,20,25,26,30,33], which hinders the attempts of directly using the envelope spectrum for bearing fault diagnosis.

Therefore, this paper proposes the FCFBI method with the following objectives:

3. To investigate and reveal the envelope spectrum characteristics of bearing vibration signals under VS;
4. To attempt to diagnose bearing faults under VS directly through envelope spectrum analysis to overcome shortcomings existing in traditional OT- and TFA-based methods.

The FCFBI method proposed in this paper is primarily based on the finding that the smeared envelope spectrum under VS contains rich bearing state information, that is, the smeared hump with the oscillating ripples [36] is related to the bearing FCFB, which can be analogized as FCF at constant speed. The range of FCFB is relevant to the fault type and rotational frequency (RF) range. Therefore, identifying FCFBs and comparing them with theoretical values allows the diagnosis of bearing faults under VS directly from the envelope spectrum.

The FCFBI method realizes automatic fault diagnosis through the following steps. First, the formula used to describe the envelope spectrum of the fault vibration signal, called the fault template, is derived from the bearing vibration signal model under VS. Then, the parameters corresponding to different faults are brought into the formula to obtain the fault templates of the inner race, outer race, and rolling element. Subsequently, within the corresponding first extended FCFB, the correlation coefficients between the measured vibration signal envelope spectrum and three different fault templates are calculated in sequence. The larger the correlation coefficient between the envelope spectrum and a certain fault template, the greater the possible that this certain fault exists in the bearing, and vice versa. According to the obtained three correlation coefficients, bearing fault diagnosis can be realized by setting a reasonable criterion. Compared with COT and TFA, FCFBI fills the gap of the state-of-the-art methods in the following aspects. Firstly, FCFBI can effectively avoid interpolation errors in COT since it does not require resampling. Secondly, the first extended FCFB is employed as the fault feature, which can prevent effects from close orders and reduce the dependence on the tachometer. Thirdly, the fault diagnosis process is simplified because FCFBI performs fault diagnosis directly through the envelope spectrum.

The originality of this paper lies in revealing the characteristics of the bearing vibration signal envelope spectrum under VS and proposing the FCFBI method for bearing fault diagnosis under VS.

The rest of this paper is organized as follows. Section 2 presents the analysis of the characteristics and the formula derivation of the envelope spectrum based on the bearing fault model under VS. The detailed FCFBI method is introduced in Section 3. In Section 4, two test bench experiments are performed to validate the proposed method. A comparative analysis of FCFBI and the other two methods followed by discussion is given in Section 5. Section 6 finally concludes this paper.

## 2. Envelope Spectrum Characteristics of Bearing Fault Vibration Signals under VS

In order to verify the feasibility of fault diagnosis by directly identifying FCFB in the envelope spectrum of the bearing vibration signal, this section proposes an REB fault vibration signal model under VS. The characteristics of the envelope spectrum are analyzed through simulation signals under different RF conditions. Meanwhile, the reason for the formation of FCFB in the envelope spectrum is deduced by the formula derivation from the model.

### 2.1. Bearing Vibration Signal Model under VS

Ref. [1] gives the vibration signal model of REB at constant speed:

(1) $$x(t)=\sum_{i=1}^{N}h(t-iT-\tau_{i})q(iT)A_{i}+n(t)$$

*N* is the total number of fault impulses. *h*(*t* – *iT* − *τ_i_*) represents the response generated by the *i*th fault impulse. *T* is the time interval between two adjacent fault impulses, *τ_i_* ∈ [0.01,0.02]*T_t_*/*N* represents the time error of the fault impulses caused by the random sliding of rolling elements, *T_t_* is the total duration of the signal. *q*(*iT*) represents the amplitude modulation of the fault impulse caused by load distribution. *A_i_* represents the amplitude uncertainty of the *i*th fault impulse. *n*(*t*) refers to noise interference generated by other vibration sources.

Compared with the constant speed condition, the main difference of the vibration signal model under VS is that the time interval between two adjacent fault impulses is not constant. Therefore, based on the model in Equation (1), a bearing vibration signal model under VS is proposed:

(2) $$x(t)=\sum_{i=1}^{N}h(t-T_{i}-\tau_{i})q(T_{i})A_{i}+n(t)$$

where *T_i_* is the arrival time of the *i*th impulse. The relationship between *T_i_* and the rotational frequency *f_r_*(*t*) (in Hz) can be expressed by

(3) $$\int_{0}^{T_{i}}f_{r}(t)dt=\frac{i}{FCC},i=1,2,\cdots ,N$$

the integration of *f_r_*(*t*) on the left is to obtain the number of bearing turns at time *T_i_*; the right side *i*/*FCC* represents the number of bearing turns at the *i*th fault impulse time instant. The value of FCC is only related to the bearing geometric parameters. The FCC of the inner race, outer race, rolling element, and cage fault can be calculated as follows:

(4) $$\left\{\begin{array}{l} FCC_{i}=\frac{z}{2}\left(1+\frac{d}{D}\cos\phi \right)\\ FCC_{o}=\frac{z}{2}\left(1-\frac{d}{D}\cos\phi \right)\\ FCC_{r}=\frac{D}{2d}\left(1-{\left(\frac{d}{D}\cos\phi \right)}^{2}\right)\\ FCC_{c}=\frac{1}{2}\left(1-\frac{d}{D}\cos\phi \right) \end{array}\right.$$

where *d* and *D* are the pitch diameter and outside diameter of the bearing, respectively. *ϕ* is the contact angle. *z* is the number of rolling elements. When *f_r_*(*t*) is known, *FCC* can be inserted into Equation (3), and then *T_i_* can be solved by the numerical method. For the bearing vibration signal under VS, the model in Equation (2) can be expressed in detail:

(5) $$x(t)=q(t)\sum_{i=1}^{N}A_{i}\underset{h(\cdot )}{\underset{︸}{e^{-\beta \left(t-T_{i}-\tau_{i}\right)}\sin\left[2\pi \omega_{r}\left(t-T_{i}-\tau_{i}\right)\right]u\left(t-T_{i}-\tau_{i}\right)}}+n(t)$$

where *q*(*t*) = 1 + *α*cos(2*π*∫fm(t)dt). *f_m_*(*t*) is the modulate frequency, which equals *f_r_*(*t*), *FCC_c_f_r_*(*t*), and 0 for inner race, rolling element, and outer race fault, respectively. Generally, the fault impulse amplitude increases with RF. This paper assumes that the amplitude of the fault impulse changes linearly with RF [24], that is, *A_i_* = *A*_0_ + *ηf_r_*(*T_i_*). *A*_0_ is a constant, and *η* is a proportional coefficient. *β* is the damping coefficient determined by the system structure. *ω_r_* is the resonance frequency of the system. *u*(·) represents the unit step function.

### 2.2. Characteristic Analysis of the Envelope Spectrum Based on Simulation Signals

Taking the parameters into Equation (5), the fault simulation signals of the bearing under VS can be obtained. Ten different *f_r_*(*t*) values are set to simulate the actual working conditions as much as possible. The detailed parameters used in Equation (5) are shown in Table 1.

The main steps of the envelope spectrum analysis include envelope demodulation and Fourier transform. Hilbert transform (HT) is the commonly used method for envelope demodulation. The HT of signal *x*(*t*) is

(6) $$H[x(t)]=\tilde{x}(t)=\pi^{-1}\int_{-\infty }^{\infty }\frac{x(\tau )}{t-\tau }d\tau$$

Construct an analytic signal with $\tilde{x}(t)$ as the imaginary part:

(7) $$X(t)=x(t)+j\tilde{x}(t)$$

where *j* is the imaginary unit. The modulus of *X*(*t*), i.e., |*X*(*t*)|, is the signal envelope. The envelope spectrum of signal *x*(*t*) can be obtained by implementing fast FT on |*X*(*t*)|.

Ten different *f_r_*(*t*) values are set to simulate the actual working conditions as much as possible. The variation modes of the first six *f_r_*(*t*) are different while their ranges remain unchanged, that is, [min(*f_r_*(*t*)),max(*f_r_*(*t*))] = [35,47] Hz. The last four *f_r_*(*t*) vary linearly while their ranges are different. The formula of these ten *f_r_*(*t*) are listed in Table 2.

**Figure 1:**
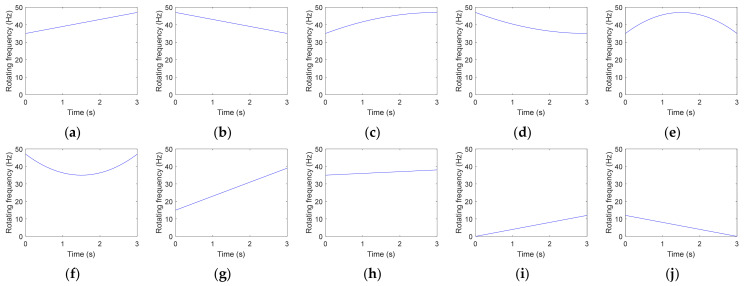
RF curves with different *f_r_*(*t*) values. (**a**–**j**) *f_r_*_1_(*t*) to *f_r_*_10_(*t*).

Corresponding RF curves are shown in Figure 1.

#### 2.2.1. Analysis under Different RF Variation Modes

**Figure 2:**
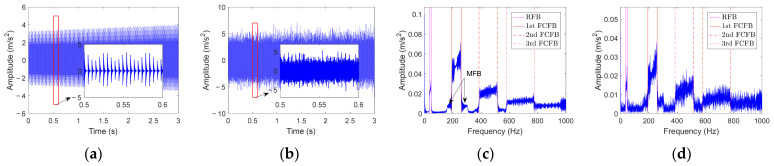
Inner race fault simulation signal with *f_r_*_1_(*t*) and its envelope spectrum. (**a**) Fault impulse signal; (**b**) Noisy impulse signal; (**c**) Envelope spectrum of the fault impulse signal; (**d**) Envelope spectrum of the noisy impulse signal.

First, the inner race fault simulation signal under *f_r_*_1_(*t*) is analyzed. Figure 2a displays the impulse signal caused by the inner race fault. The local magnified view of the red box area shows that the impulse amplitude is modulated by time-varying RF. The curve shown in Figure 2b is the signal with −5 dB Gaussian white noise added to the fault impulse, which is submerged by noise. Figure 2c is the envelope spectrum corresponding to Figure 2a. There are three distinct smeared humps with oscillating ripples in the low frequency band. The frequency ranges of these three humps are marked with a red solid line, dotted line, and dotted–dashed line, i.e., *FCC_i_* × [*f_r_*_1_(0), *f_r_*_1_(3)], 2*FCC_i_* × [*f_r_*_1_(0), *f_r_*_1_(3)], and 3*FCC_i_* × [*f_r_*_1_(0), *f_r_*_1_(3)], respectively. Since the endpoint frequency of these humps is related to bearing FCF and its harmonics at the lowest RF and the highest RF, this paper refers to these peaks as the fault characteristic frequency band, or FCFB. As the harmonic increases, the amplitude of FCFB tends to decay until all FCFBs are connected together without significant boundaries. In addition to the FCFBs in the envelope spectrum, there is also a frequency band related to RF, marked with magenta solid lines, called the rotational frequency band (RFB), which ranges from *f_r_*_1_(0) to *f_r_*_1_(3). Meanwhile, distinct time-varying RF sidebands, called modulation frequency bands (MFBs), appear on both sides of the 1st FCFB. Figure 2d illustrates the envelope spectrum corresponding to Figure 2b. Its main characteristics are consistent with those in Figure 2c. Although affected by noise, the FCFBs that can reflect the fault characteristics of the inner race still appear clearly in the envelope spectrum.

**Figure 3:**
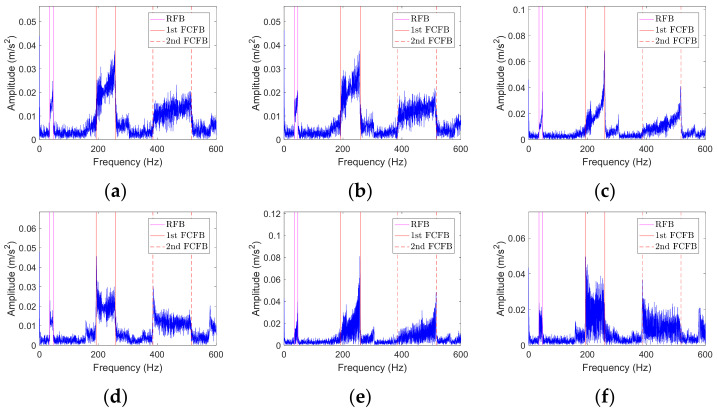
Envelope spectra of the noisy inner race fault simulation signal with different RF modes. (**a**–**f**) *f_r_*_1_(*t*) to *f_r_*_6_(*t*).

Then, the noisy inner race fault simulation signal with *f_r_*_2_(*t*) to *f_r_*_6_(*t*) is analyzed sequentially. The corresponding envelope spectra in the 0–600 Hz frequency band are depicted in Figure 3b–f. At the same time, the envelope spectrum of the signal with *f_r_*_1_(*t*) is also depicted in Figure 3a for comparative analysis. It is obvious that the 1st FCFB and the 2nd FCFB shown in Figure 3b–f have the same range as that in Figure 3a. Meanwhile, RFBs also appears in interval [min(*f_ri_*(*t*)), max(*f_ir_*(*t*))], (*i* = 2, …, 6). However, it can be found that the amplitude variation trends inside the FCFB are not the same. Through the comparison of Figure 3a,b, it is found that when RF linearly increases and decreases, the amplitude variation trends inside the FCFB are almost the same. Based on a comparison of Figure 3c with Figure 3a, although both RF values increase from 35 Hz to 47 Hz, the rising trend of the amplitude inside FCFB is more intense when RF increases non-linearly. Figure 3d shows the envelope spectrum with the non-linear decreasing RF. The amplitude in FCFB generally presents a decreasing trend. Figure 3e,f reveal the envelope spectrum when RF increases then decreases and decreases then increases non-linearly, respectively. The amplitude trends in FCFB are similar to those in Figure 3c,d, but the oscillation is more intense.

Through the above simulation signal analysis, it can be concluded that when RF ranges, that is, [min(*f_r_*(*t*)), max(*f_r_*(*t*))] remain unchanged, the ranges of FCFB will remain the same. The RF variation mode only affects the amplitude variation inside the FCFB.

#### 2.2.2. Analysis under Different RF Variation Ranges

Large and small RF variation ranges are very common for bearings in actual operation. When max(*f_r_*(*t*)) > 2 min(*f_r_*(*t*)), the ending frequency of the 1st FCFB will be greater than the starting frequency of the 2nd FCFB, so the first two FCFBs will overlap. Therefore, in this paper, the RF range that meets max(*f_r_*(*t*)) > 2 min(*f_r_*(*t*)) is defined as the large RF variation range, while the small RF variation range occurs when it meets max(*f_r_*(*t*))/min(*f_r_*(*t*)) ≤ 1.2. Start up and coast down are two common working conditions of rotating machinery. Under these two working conditions, the bearing RF will increase from 0 Hz to the rated frequency and decrease from nominal frequency to 0 Hz. Since it has been concluded that the range of FCFB is independent of the RF variation mode, this section sets *f_r_*(*t*) to the linear variation mode and only changes the ranges. The formulas of *f_r_*_7_(*t*) to *f_r_*_10_(*t*) are also listed in Table 2, and corresponding curves are shown in Figure 1g–j.

**Figure 4:**
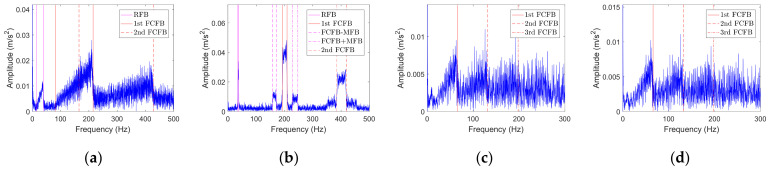
Envelope spectra of the noisy inner race fault simulation signal with different RF ranges. (**a**–**d**) *f_r_*_7_(*t*) to *f_r_*_10_(*t*).

Figure 4a illustrates the envelope spectrum of the inner race fault noisy signal under a large RF variation range. Although contaminated by noise, RFB and the 1st FCFB can be clearly identified in the low frequency band. Since the first two FCFBs are overlapped, the starting point of the 2nd FCFB is submerged in the 1st FCFB. However, due to superposition effects, there is a small jump in the envelope spectrum magnitude at the start of the 2nd FCFB. In contrast, the endpoint of the 2nd FCFB is clearly visible. The envelope spectrum of the inner race fault noisy signal under the small RF variation range is displayed in Figure 4b. It can be observed that significant FCFBs appear in the envelope spectrum. For the 1st FCFB, distinct MFBs appear on its both sides, that is, FCFB ± MFB. The corresponding left sideband range is (*FCC_i_* − 1) × [min(*f_r_*_8_(*t*)), max(*f_r_*_8_(*t*))]. The right sideband range is (*FCC_i_* + 1) × [min(*f_r_*_8_(*t*)), max(*f_r_*_8_(*t*))]. Similarly, for the *n*th FCFB, the corresponding left sideband range is equal to (*nFCC_i_* − 1) × [min(*f_r_*_8_(*t*)), max(*f_r_*_8_(*t*))] and the right sideband range is equal to (*nFCC_i_* + 1) × [min(*f_r_*_8_(*t*)), max(*f_r_*_8_(*t*))]. Figure 4c,d are the envelope spectrum of the inner race fault noisy signal with RF increasing from 0 Hz and decreasing to 0 Hz, respectively. It can be found that the envelope spectra in both cases are very similar. The starting points of the first three FCFBs are 0 Hz. The red lines indicate the terminal frequencies of FCFBs, which can be clearly distinguished, i.e., there are amplitude jumps near the terminal points of the corresponding FCFBs. However, covered by the 1st FCFB, RFB is difficult to distinguish.

The envelope spectrum analysis of the inner race fault simulation signal with different RF variation range further verifies that the range of FCFB under the same fault type is related only to the RF range.

#### 2.2.3. Analysis under Different Fault Types

**Figure 5:**
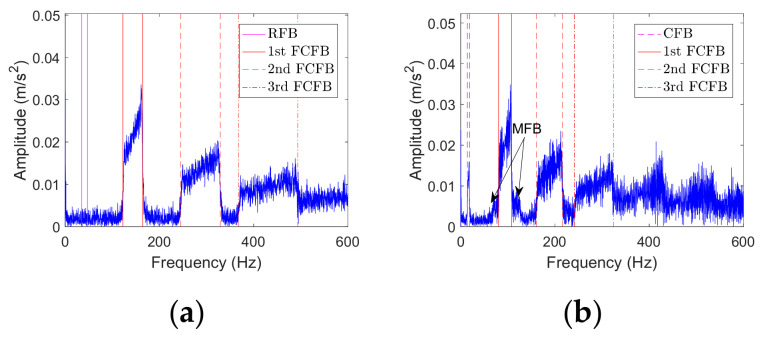
Envelope spectra of the noisy outer race and rolling element fault simulation signal. (**a**) Outer race fault; (**b**) Rolling element fault.

In this section, envelope spectrum analysis will be performed on the noisy outer race fault and rolling element fault simulation signals under the same RF, i.e., *f_r_*_1_(*t*). Figure 5a displays the envelope spectrum of the outer race fault simulation signal. The 1st FCFB appears in the frequency band *FCC_o_* × [*f_r_*_1_(0), *f_r_*_1_(3)], and the 2nd FCFB and the 3rd FCFB are clear. RFB and MFB do not appear since there is no RF modulation for the outer race fault. The envelope spectrum of the rolling element fault simulation signal is depicted in Figure 5b. The interval of the 1st FCFB is *FCC_r_* × [*f_r_*_1_(0), *f_r_*_1_(3)], and the 2nd FCFB and the 3rd FCFB are also distinct. MFB also appears in the envelope spectrum due to modulation by the cage frequency. The frequency band marked by magenta dotted lines is related to the cage frequency, which is called the cage frequency band (CFB). Combining Figure 5 and Figure 2d, it is clear that FCFBs exist in the envelope spectrum of the bearing signal under different fault types. The range of FCFBs varies with fault types, while the amplitude trends inside the FCFBs basically remain the same.

### 2.3. Formula Derivation of the Envelope Spectrum under VS

In this section, based on the derivation of the envelope spectrum formula, the reason for the formation of FCFB is theoretically investigated to verify the feasibility of FCFBI. FCFB exists in the envelope spectrum regardless of fault types and can directly reflect the fault type of the bearing. While RFB only exists in the inner race fault envelope spectrum, MFB does not exist in the outer race fault envelope spectrum. Meanwhile, the amplitude of MFB on both sides of FCFB is low. Therefore, in order to simplify the analysis process, for the model in Equation (5), the amplitude modulation caused by the inner race or rolling element fault, the uncertainty of the fault impulse amplitude caused by RF variation, and the noise are not considered. The simplified model is as below:

(8) $$x_{s}(t)=\sum_{i=1}^{N}\underset{\mathrm{fault}\;\mathrm{impulse}}{\underset{︸}{e^{-\beta \left(t-T_{i}-\tau_{i}\right)}}}\underset{\mathrm{high}\;\mathrm{frequency}\;\mathrm{carrier}}{\underset{︸}{\sin\left[2\pi \omega_{r}\left(t-T_{i}-\tau_{i}\right)\right]}}u\left(t-T_{i}-\tau_{i}\right)$$

**Figure 6:**
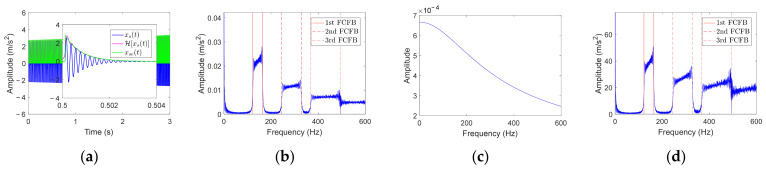
Envelope spectrum analysis based on the formula derivation. (**a**) Simplified signal and its envelope; (**b**) Curve of |*X_se_*(*jω*)|; (**c**) Curve of |1/(*β*+*jω*)|; (**d**) Curve of $\left|\sum_{i=1}^{N}e^{{j\omega (T}_{i}{+\tau }_{i})}\right|$.

Let RF be *f_r_*_1_(*t*), and bring *FCC_o_* and other parameters in Table 1 into Equation (8); the simplified fault vibration signal can be obtained, as shown by the blue curve in Figure 6a. The upper envelope calculated by HT is shown in the magenta curve in local magnified view. The green curve displays the ideal upper envelope of the fault impulse, that is,

(9) $$x_{se}(t)=\sum_{i=1}^{N}e^{-\beta \left(t-T_{i}-\tau_{i}\right)}u\left(t-T_{i}-\tau_{i}\right)$$

It can be seen that curve ℋ[*x_s_*(*t*)] and basically coincides with *x_se_*(*t*), so we can approximate *x_se_*(*t*) as the Hilbert envelope of *x_s_*(*t*). Consequently, the envelope spectrum of *x_s_*(*t*) can be derived by implementing FT in *x_se_*(*t*), that is,

(10) $$X_{s}(jw)\approx X_{se}(jw)=F[x_{se}(t)]=\frac{1}{\beta +j\omega }\sum_{i=1}^{N}e^{-j\omega (T_{i}+\tau_{i})}$$

where *ω* = 2*πf*; *f* is the frequency in Hertz. The curve of |*X_se_*(*jω*)|, calculated by modulo the right side of Equation (10), is shown in Figure 6b. FCFBs related to the outer race fault can be observed and have the same range as FCFBs obtained from the envelope spectrum of the outer race fault simulation signal in Figure 5a. Moreover, with *f* as the abscissa, |1/(*β* + *jω*)| and $\left|\sum_{i=1}^{N}e^{{j\omega (T}_{i}{+\tau }_{i})}\right|$ are functions of *f*. The corresponding curves are plotted in Figure 6c,d. In Figure 6d, FCFBs have the same range with those in Figure 5a and Figure 6b, while the decaying trend of FCFBs in Figure 6d is slower. Figure 6c depicts a monotonic decreasing curve, which is independent of FCFB. Therefore, from the envelope spectrum formula, we can deduce that the formation of FCFBs in the envelope spectrum is related to *T_i_*+*τ_i_* in $\left|\sum_{i=1}^{N}e^{{j\omega (T}_{i}{+\tau }_{i})}\right|$. As the value of *τ_i_* is very small compared with *T_i_*, it can be concluded that the fault impulse with unequal interval *T_i_* in the time domain is the root reason of the formation of FCFBs in the bearing envelope spectrum under VS, which theoretically proves that FCFBs in the envelope spectrum can be employed for bearing fault diagnosis under VS.

Based on the above simulation and theoretical analysis, the following conclusions can be drawn:

- Conclusion 1: Regular FCFBs appear in the low frequency band of the envelope spectrum of the bearing vibration signal under VS. The range of the *n*th FCFB is *nFCC* × [min(*f_r_*(*t*)),max(*f_r_*(*t*))]. The amplitude of FCFB shows a decaying trend from the 1st to the *n*th FCFB. MFBs exist in the inner race and rolling element fault envelope spectrum, and their ranges around the *n*th FCFB are *n*(*FCC_i_* ± 1) × [min(*f_r_*(*t*)), max(*f_r_*(*t*))] and *n*(*FCC_r_* ± *FCC_c_*) × [min(*f_r_*(*t*)), max(*f_r_*(*t*))], respectively. At the same time, RFB and CFB exist in the inner race and rolling element fault envelope spectrum, and their ranges are equal to the range of RF and the cage frequency, respectively.
- Conclusion 2: The fundamental reason why FCFB in the envelope spectrum lies in the fault impulse is the unequal time interval in the time domain under VS. The range of FCFB is related to the fault type and RF range rather than the variation mode of RF, which affects only the amplitude inside the FCFB.
- Conclusion 3: FCFB, as an important characteristic of the bearing fault vibration signal envelope spectrum under VS, can be utilized for fault diagnosis.

## 3. The Proposed FCFBI Bearing Fault Diagnosis Method under VS

In practical applications, due to the vibration of other components in rotating machinery and the interference of external noise, it is difficult to perform fault diagnosis by manually observing the FCFB in the envelope spectrum. In this section, a novel method that can automatically identify FCFB and is less susceptible to noise, i.e., FCFBI, is proposed for bearing fault diagnosis under VS.

### 3.1. Correlation Coefficient-Based FCFBI

The key to accurate identification of FCFB is to find the amplitude jump near the starting and end points of FCFB. The amplitude of the envelope spectrum at FCFB tends to attenuate as the harmonic order increases. The 1st FCFB is the easiest to identify in all FCFBs because the sudden change in amplitude at the endpoints is most pronounced. Since energy leakage in the FFT calculation is inevitable, when identifying the 1st FCFB, it is necessary to extend the interval of the 1st FCFB to a small range *ex*, i.e., [*FCC* × min(*f_r_*(*t*)) − *ex*, *FCC* × max(*f_r_*(*t*)) + *ex*], we denote the extended FCFB as *FCFBex*. Inside *FCFBex*, the amplitude presents a sharp rise around *FCC* × min(*f_r_*(*t*)), then oscillates inside FCFB, and finally falls sharply around *FCC* × max(*f_r_*(*t*)). Under the same RF, if bearing fault types are different, the frequency band position of the 1st FCFB in the envelope spectrum will be different. Therefore, if $\left|\sum_{i=1}^{N}e^{{j\omega (T}_{i}{+\tau }_{i})}\right|$ is introduced as the fault template, the ideal envelope spectra under the inner race, outer race, and rolling element fault can be obtained. The next steps are to intercept the templates of different faults in the corresponding *FCFBex* as comparison references and to then calculate the correlation coefficients between these references and the actual signal envelope spectrum in the corresponding *FCFBex*. These obtained correlation coefficients can reflect the possibility of different fault-related FCFBs appearing in the bearing signal envelope spectrum, that is, the possibility of different types of fault existing in the bearing. By comparison with the preset correlation coefficient range, automatic bearing fault diagnosis under VS can be realized.

**Figure 7:**
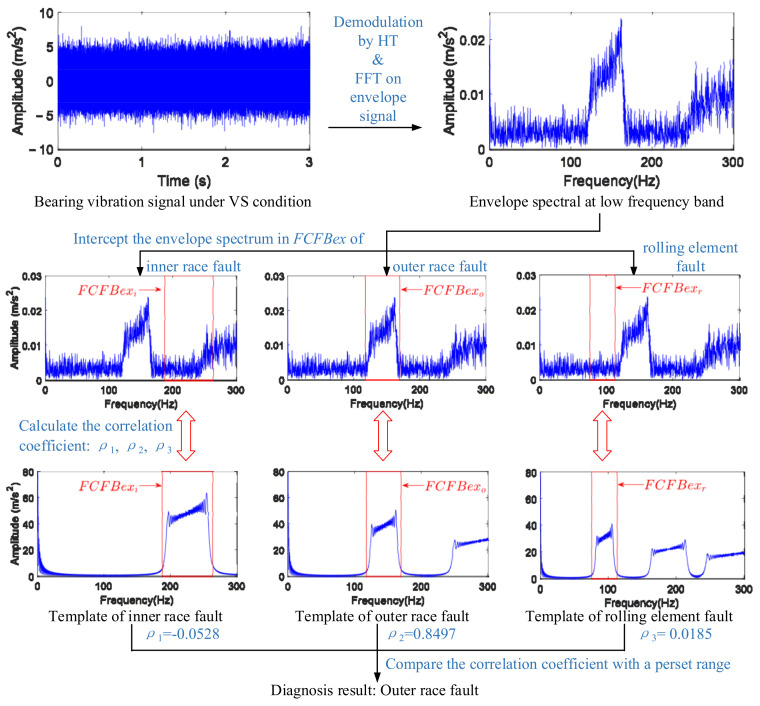
Schematic of the correlation coefficient-based FCFBI fault diagnosis method.

Figure 7 takes the outer race fault simulation signal with *f_r_*_1_(*t*) and −10 dB SNR as an example, illustrating the schematic of the aforementioned correlation coefficient-based FCFBI bearing fault diagnosis method. In this example, the correlation coefficient between the envelope spectrum in *FCFBex_o_* and the outer race fault template is greater than 0.8, while the correlation coefficient the other two templates is close to 0, so the diagnosis result of the bearing outer race fault can be obtained.

### 3.2. FCFB Feature Enhancement

**Figure 8:**
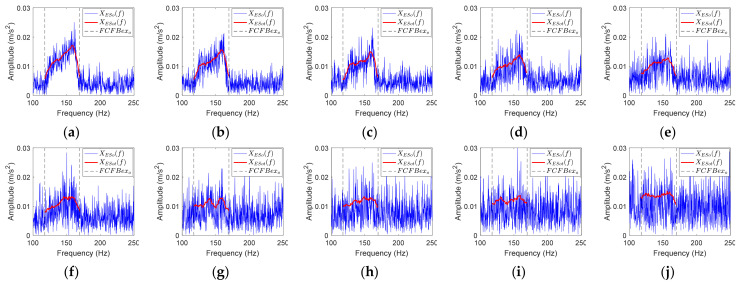
The envelope spectra of the noisy outer race fault simulation signal under different SNRs. (**a**–**j**) SNR decreases from −11 dB to −20 dB with a 1 dB step.

**Figure 9:**
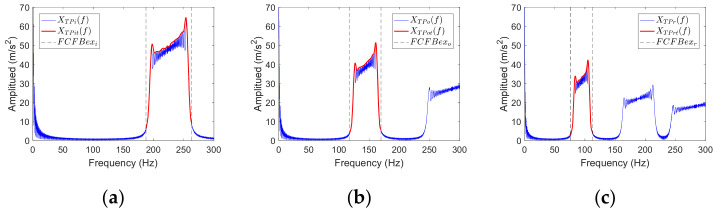
Fault templates. (**a**) Inner race fault; (**b**) Outer race fault; (**c**) Rolling element fault.

Bearing vibration signals are susceptible to noise interference, which will affect the accuracy of the correlation coefficient-based FCFBI fault diagnosis method. The blue curves in Figure 8 display the envelope spectrum of the outer race fault simulation signal with different noise levels. It can be noticed that the 1st FCFB is clear when SNR = −11~−15 dB (Figure 8a–e), while the 1st FCFB is masked by noise when SNR = −16~−20 dB (Figure 8f–j). Furthermore, as the SNR decreases, the amplitude jumps near both ends of FCFB become blurred, and the amplitude oscillation intensifies inside the FCFB. The blue curve in Figure 9 shows the different fault templates. The amplitude of the template oscillation in FCFB can be seen, which will also influence the accuracy of the correlation coefficient-based FCFBI. Therefore, it is necessary to enhance the FCFB feature before calculating the correlation coefficient.

The main principle of the correlation coefficient-based FCFBI method is to compare the consistency between the envelope spectrum trend with the fault template trend in the corresponding *FCFBex*. Hence, if the trend curves can be fitted from the both envelope spectrum of the original signal and the fault templates in *FCFBex*, the correlation coefficient can be calculated between these trend curves instead of the original curves. The interference on the correlation coefficient accuracy caused by the signal noise and template amplitude oscillation will be alleviated after trend fitting. Consequently, the fault diagnostic accuracy can be improved.

#### 3.2.1. Envelope Spectrum FCFB Trend Fitting

From the envelope spectrum of the noisy signal in Figure 8, we can see that the peak-to-peak amplitudes in interval [0, *FCC* × min(*f_r_*(*t*))] and [*FCC* × max(*f_r_*(*t*)), 2*FCC* × min(*f_r_*(*t*))] are close to that inside the 1st FCFB. The 1st FCFB is characterized by an overall increase in amplitude in interval *FCC* × [min(*f_r_*(*t*)), max(*f_r_*(*t*))], that is, the energy of the envelope spectrum in the 1st FCFB is higher than that in other intervals. Since the root mean square (RMS) can reveal the energy level of the signal, FCFB trend fitting can be done by computing the RMS value of the envelope spectrum using a sliding window with length *wl*. Regarding the obtained RMS sequence as the trend of FCFB can reduce the influence of noise. The steps of the envelope spectrum FCFB trend fitting based on the RMS sliding window are as follows:

1. Denote the envelope spectrum of the bearing vibration signal as *X_ES_*(*f*); the corresponding inner race, outer race, and rolling element fault signal envelope spectra are denoted as *X_ESi_*(*f*), *X_ESo_*(*f*), and *X_ESr_*(*f*), respectively. Assuming that *X_ES_*(*f*) contains *m* data points, remove the DC component to obtain the centered envelope spectrum: *X_c_*(*f*) = *X_ES_*(*f*) − *mean*(*X_ES_*(*f*));
2. For the *i*th data point *X_c_*(*f*)*_i_* (*i* = 1, …, *m*), calculate the RMS value of this point and the previous *wl* − 1 points to obtain $X_{RMS}{\left(f\right)}_{i}=\left\{\begin{array}{l} X_{c}{\left(f\right)}_{i}+\sqrt{\frac{1}{n}\sum_{i=1}^{n}{\left|X_{c}{\left(f\right)}_{i}\right|}^{2}},\;n<wl\\ X_{c}{\left(f\right)}_{i}+\sqrt{\frac{1}{wl}\sum_{i=n-wl+1}^{n}{\left|X_{c}{\left(f\right)}_{i}\right|}^{2}},\;n\geq wl \end{array}\right.$;
3. Intercept *X_RMS_*(*f*) in *FCFBex* to obtain the trend of *X_ES_*(*f*), which is denoted as *X_ESt_*(*f*). Similarly, the trends of the envelope spectra under different faults are denoted as *X_ESti_*(*f*), *X_ESto_*(*f*), and *X_EStr_*(*f*).

The red curves in Figure 8 indicate the trend of the envelope spectrum in *FCFBex* obtained by the above trend fitting method when *wl* = 50. It can be noticed that when SNR = −11~−16 dB, the amplitude jumps at the endpoints of the 1st FCFB are more distinct after trend fitting. At the same time, the amplitude oscillations in *FCFBex* of all simulation signals are alleviated.

#### 3.2.2. Template FCFB Trend Fitting

Since fault templates are noise free, the 1st FCFB in template is very clear in Figure 9. Nevertheless, small amplitude oscillation still appears inside the 1st FCFB, which will also influence the correlation coefficient. If the same FCFB trend fitting method as the envelope spectrum is adopted here, the amplitude jumps at the endpoints of the 1st FCFB will be suppressed, while the low amplitudes at other places in the template will increase. This will weaken the feature of FCFB in the template. The highest point of the template near the amplitude jump is the local extreme point of FCFB. Inside FCFB, there also exist multiple local extreme points. Finding all of these extreme points and selecting and interpolating between them are steps to obtain a substitute for the original template. Then, the amplitude oscillation in FCFB will be eliminated. The template FCFB trend fitting steps based on the interpolation of the local extreme points are introduced as follows:

1. Denote fault template as *X_TP_*(*f*); the corresponding inner race, outer race, and rolling element fault templates are denoted as *X_TPi_*(*f*), *X_TPo_*(*f*), and *X_TPr_*(*f*), respectively. Find all local extreme points of *X_TP_*(*f*) in *FCFBex*; record these values as set {*m_k_* (*k* = 1, …, *n*)}, with the corresponding location in *FCFBex* as {*Loc_k_* (*k* = 1, …, *n*)};
2. Let *l* = 0, and denote the minimum peak distance between local extreme points as *mpd*;
3. Find the maximum value in {*m_k_*}, i.e., *m_kmax_* = max(*m_k_*), and its corresponding location *Loc_kmax_*;
4. Ignore the local extreme points located in [*Loc_kmax_* − *mpd*, *Loc_kmax_* + *mpd*] and update the remaining set of extreme points as {*m_k_* (*k* = 1, …, *n_update_*)};
5. If *n_update_* > 1, let *l* = *l* + 1, repeat steps 2–4;
6. Obtain all of the updated sets of the extreme points {*m_kmax_* (*kmax* = 1, …, *l*)} and the corresponding location set {*Loc_kmax_* (*kmax* = 1, …, *l*)}. Treat (*Loc_kmax_*, *m_kmax_*) as original values and use cubic spline interpolation to obtain the value of other locations in *FCFBex*.

In step 2, *mpd* is set to control the smoothness of the template. Denote the trends of *X_TPi_*(*f*), *X_TPo_*(*f*) and *X_TPr_*(*f*) as *X_TPit_*(*f*), *X_TPot_*(*f*), and *X_TPrt_*(*f*). The red curves in Figure 9 illustrate the fitted trend of templates in *FCFBex* when *mpd* = 10. It can be perceived that the characteristics of FCFB in templates are preserved while the amplitude oscillations are removed effectively.

### 3.3. Procedure of the FCFBI Method

**Figure 10:**
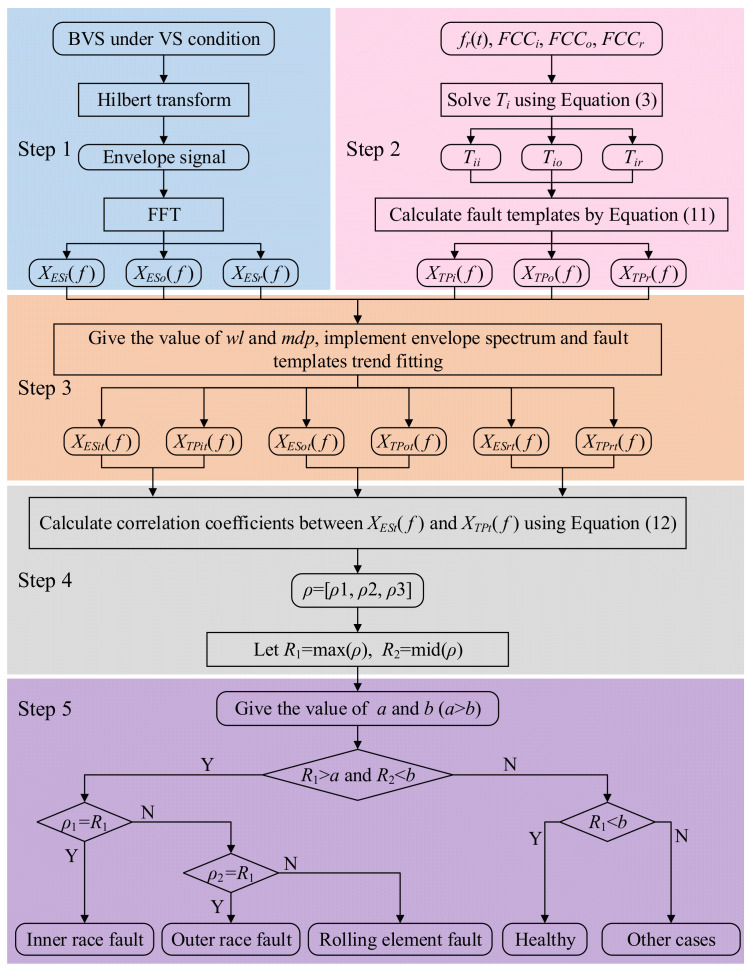
Flowchart of the proposed FCFBI method.

Combining the aforementioned correlation coefficient-based FCFBI and feature enhancement methods, the flowchart of the REB fault diagnosis method based on FCFBI under VS is illustrated in Figure 10. The specific steps of the FCFBI methods are listed as follows:

1. Perform HT on the bearing vibration signal (BVS) under VS to obtain the envelope signal. Then, carry out FFT on the envelope signal to determine the envelope spectrum. Intercept the envelope spectrum in *FCFBex* to obtain *X_ESi_*(*f*), *X_ESo_*(*f*), and *X_ESr_*(*f*);
2. Insert *f_r_*(*t*), *FCC_i_*, *FCC_o_*, and *FCC_r_* into Equation (3), and then the impulse occurrence time *T_i_* of the inner race, outer race, and rolling element fault can be solved, i.e., *T_ii_*, *T_io_*, and *T_ir_*. Bring them into
  (11) $$X_{TP}(f)=\left|\sum_{i=1}^{N}e^{-j2\pi f(T_{i}+\tau_{i})}\right|$$
  to obtain different fault templates;
3. Find the value of *wl*, fit the envelope spectrum in FCFBs by the sliding window RMS method to obtain *X_ESit_*(*f*), *X_ESot_*(*f*), and *X_ESrt_*(*f*) in the corresponding *FCFBex*. At the same time, *X_TPit_*(*f*), *X_TPot_*(*f*), and *X_TPrt_*(*f*) can be calculated using the local extreme point interpolation method with given *mpd*;
4. In *FCFBex*, utilize
  (12) $$\rho_{X_{ES},X_{IP}}=\frac{E\left[\left(X_{ES}-\mu_{X_{ES}}\right)\left(X_{TP}-\mu_{X_{IP}}\right)\right]}{\sigma_{X_{ES}}\sigma_{X_{TP}}}$$
  to calculate the correlation coefficients between *X_ESit_*(*f*) and *X_TPit_*(*f*), *X_ESot_*(*f*) and *X_TPot_*(*f*), and *X_ESrt_*(*f*) and *X_TPrt_*(*f*), i.e., *ρ*_1_, *ρ*_2_, and *ρ*_3_. In Equation (12), E, *μ,* and *σ* indicate the calculated expectation, mean value, and variance, respectively. Let *ρ* = [*ρ*_1_, *ρ*_2_, *ρ*_3_], *R*_1_ = max(*ρ*), *R*_2_ = mid(*ρ*);
5. Preset *a* and *b* (*a*,*b* ∈ [−1,1], *a* > *b*) as the upper and lower limits of the correlation coefficients. The values of *a* and *b* can be determined with trial and error when bearing fault signal samples can be obtained in advance. Output the diagnostic result according to the judgment rule at the bottom side of Figure 10. Other cases may include compound faults, which will not be studied in this paper.

**Figure 11:**
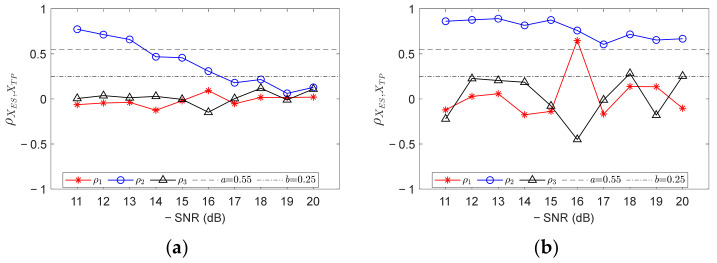
Diagnostic results of outer race fault simulation signals under different SNRs. (**a**) Result without trend fitting; (**b**) Result with trend fitting.

In order to verify the effect of FCFB feature enhancement on improving the diagnostic accuracy, the proposed FCFBI method is applied to analyze the simulation signals shown in Figure 8. Figure 11a shows the correlation coefficients between envelope spectra and different fault templates. The red asterisk, blue circle, and black triangle marks represent the respective correlation coefficients between the envelope spectrum and inner race, outer race, and rolling element fault template in the corresponding *FCFBex*. It can be observed that the correlation coefficients between the envelope spectrum and outer race fault template, i.e., *ρ*_2_, present a downward trend as SNR decreases. Given *a* = 0.55 and *b* = 0.25, only the simulation signal with SNR = −11~−13 dB can be diagnosed as the outer race fault. Figure 11b displays the correlation coefficients after the trend fitting of the envelope spectra and fault templates, where *mpd* = 10, *wl* = 50, *ex* = 5 Hz. It can be found that under all SNRs, the values of all *R*_1_ are higher than *a*, and there is no obvious decline with the decrease in SNR. The simulation signals with SNR = −11~−15 dB, −17 dB, and −19 dB can be identified as the outer race fault. The diagnostic accuracy improved compared to results without trend fitting.

## 4. Experiment Validation

In this section, two sets of bearing vibration experiments under VS are carried out to validate the proposed FCFBI fault diagnosis method. The data of the first experiment come from the public bearing vibration data set, while the data of the second experiment are from the rotating machinery fault simulation test bench.

### 4.1. Experiment One: Inner Race Fault

**Figure 12:**
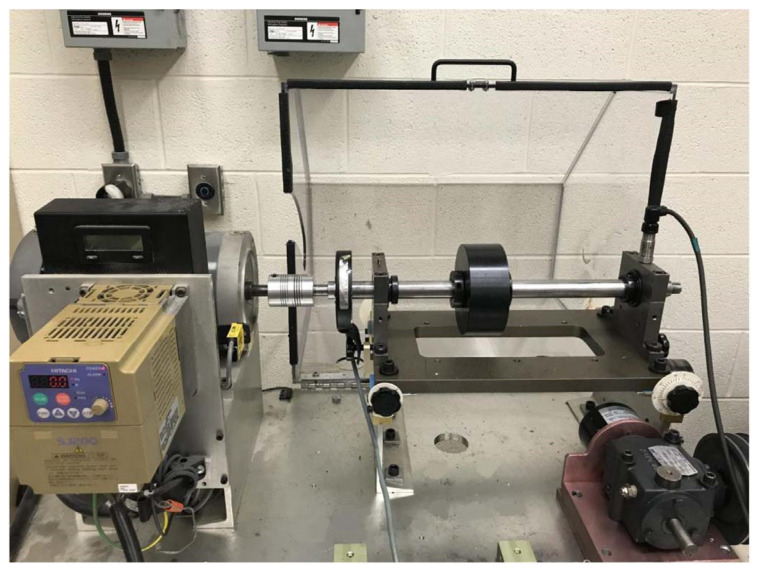
Experiment setup for the bearing inner race fault [35].

Inner race fault vibration data under VS from the University of Ottawa [37] are employed for the first validation. The data were collected from the SpectraQuest machinery fault simulator (MFS-PK5M); its structure is shown in Figure 12. The shaft is driven by a motor whose speed is adjusted by an AC drive and measured by an incremental encoder. Both sides of the shaft are supported by an ER16K ball bearing; the left bearing is in a healthy state, and the inner race fault is introduced on the right bearing. An ICP accelerometer is installed on the right bearing seat to collect the fault bearing vibration signal. According to the geometric parameters of the ER16K bearing, *FCC_i_*, *FCC_o_*, and *FCC_r_* are equal to 5.43, 3.57, and 2.32, respectively.

The sampling frequency of the original data is 200 kHz. All signals are down-sampled to 20 kHz to reduce the computational burden. The experiment is conducted under four RF variation conditions: increasing, decreasing, increasing then decreasing, and decreasing then increasing. Three trials are conducted for each experimental setting. In order to verify FCFBI more comprehensively, each data is further segmented according to the different ranges of RF, that is, small variation range satisfying max(*f_r_*) = 1.2 min(*f_r_*) and medium variation range satisfying max(*f_r_*) = 1.5 min(*f_r_*). In addition, for RF increasing and decreasing conditions, the data satisfying max(*f_r_*) > 2 min(*f_r_*) are defined as the large variation range. The details of the bearing inner race fault data set can be found in Table A1.

**Figure 13:**
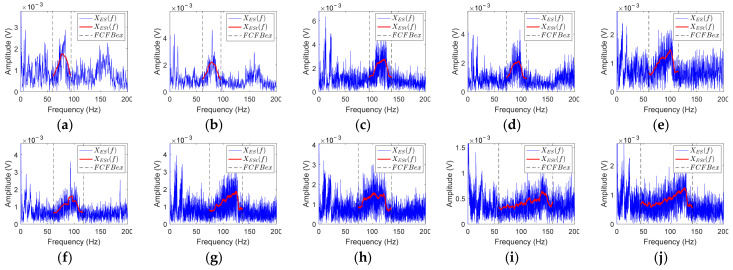
Envelope spectra of inner race fault vibration signals. (**a**) S-A-2; (**b**) S-B-2; (**c**) S-C-2; (**d**) S-D-2; (**e**) M-A-2; (**f**) M-B-2; (**g**) M-C-2; (**h**) M-D-2; (**i**) L-A-1; (**j**) L-B-1.

Taking ten sets of experimental data as examples, the corresponding vibration signal envelope spectra in the low frequency band are depicted in Figure 13. The blue curves represent the envelope spectrum of the original signal. The black dotted lines show the *FCFBex* interval corresponding to RF of each data when *ex* = 10 Hz. The envelope spectra corresponding to ranges S and M have obvious amplitude jumps near the endpoints of the 1st FCFB. However, for range L, the 1st FCFB is not significant, but amplitude jumps are apparent near the end point of the 1st FCFB and the starting point of the 2nd FCFB, which is consistent with the simulation analysis in Section 2.2.2.

**Figure 14:**
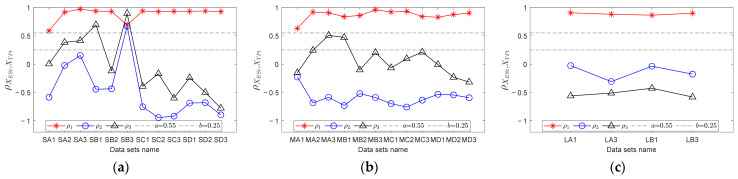
Diagnostic results of inner race fault data with trend fitting. (**a**) Small RF variation range data set; (**b**) Medium RF variation range data set; (**c**) Large RF variation range data set.

For each data point, RF curves can be computed according to the encoder signal. Then, through step 2 in Section 3.3, fault templates can be obtained. According to step 3, let *mdp* = 20, *wl* = 50, and *ex* = 10 Hz; calculate the trend curves of the envelope spectrum and fault templates in *FCFBex* sequentially. The red curves in Figure 13 show the fitted trend of envelope spectra. We can conclude that the fitting process can not only enhance the features of FCFB but can also effectively reduce the amplitude oscillation. Fault template curves will not be plotted in this paper due to space limitations. The correlation coefficients between the trend curves of the envelope spectrum and fault templates in the corresponding *FCFBex* are computed in step 4. In step 5, we set the upper and lower limits of the correlation coefficient as *a* = 0.55 and *b* = 0.25. Figure 14a illustrates the diagnostic results of small RF variation range data. Except for S-A-2, S-A-3, S-B-1, and S-B-3, other eight data can be diagnosed as inner race faults. The results of medium RF variation range data are displayed in Figure 14b. Among twelve data, except for M-A-3 and M-B-1, ten other data can be identified as inner race faults. The correlation coefficients presented in Figure 14c imply that all four data with a large RF variation range are successfully diagnosed.

**Figure 15:**
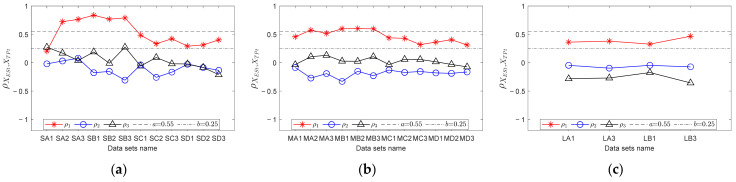
Diagnostic results of inner race fault data without trend fitting. (**a**) Small RF variation range data set; (**b**) Medium RF variation range data set; (**c**) Large RF variation range data set.

In order to validate the improvement of the diagnostic accuracy by FCFB feature enhancement, the correlation coefficients between the original envelope spectra and original fault templates were also calculated. Figure 15 depicts the corresponding results. Under the same correlation coefficient upper and lower limit settings, only S-A-2, S-A-3, S-B-1, and S-B-2 are diagnosed as inner race faults among the small RF variation range data. For the medium RF variation range data, only M-A-2, M-B-1, M-B-2, and M-B-3 can be distinguished as inner race faults, whereas no data sets under the large RF variation range are diagnosed correctly.

In summary, in the bearing inner race fault experiment, the diagnostic accuracy of FCFBI and FCFBI without trend fitting is 78.6% (22 out of 28 data) and 28.6% (8 out of 28 data), respectively. Their corresponding average correlation coefficients are 0.878 and 0.503, respectively. It is not difficult to find that trend fitting greatly improves the FCFBI-based diagnostic accuracy.

### 4.2. Experiment Two: Outer Race Faults

**Figure 16:**
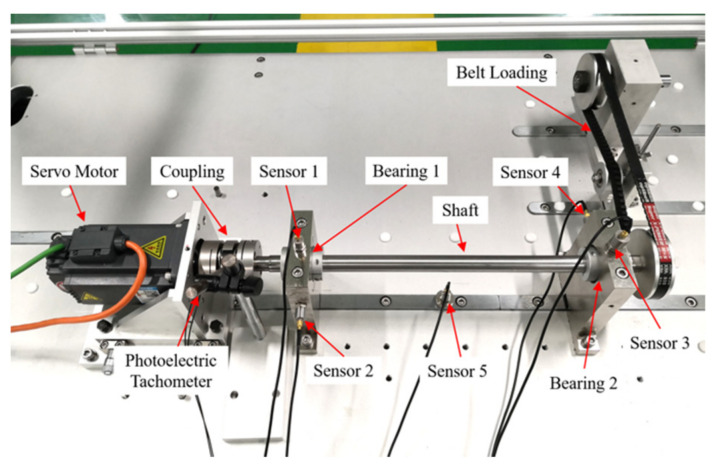
Experimental setup for bearing outer race faults.

The bearing outer race fault experimental data under VS were collected from the rotating machinery fault simulation test bench in Beijing Jiaotong University. The structure of the test bench is shown in Figure 16.

The main mechanical components of the test bench include a servo motor, a rotating shaft supported by two bearings, and a belt-loading device. The servo motor is connected to the shaft through flexible coupling, and the rotation speed is adjusted by the frequency converter. Two strips of black tape are pasted on the coupling surface to measure the shaft speed by a photoelectric tachometer. Each bearing seat is equipped with two mutually perpendicular accelerometers, i.e., sensors 1–4. At the same time, the fifth accelerometer is mounted on the base of the bench. Bearing 1 is a 6204 deep-groove ball bearing, which is set as the healthy state throughout the experiment. Bearing 2 is an NU204 cylindrical roller bearing, which is set as the outer race fault in the experiment. The fault is processed by wire-electrode cutting. The shape of the defect is a rectangular groove along the axial direction of the outer ring, with width of 0.7 mm and depth of 0.5 mm. The geometric parameters of the NU204 bearing and the calculated FCF are listed in Table 3.

The RF variation mode of the outer race fault experiment includes six conditions: increasing, decreasing, increasing then decreasing, decreasing then increasing, increasing from 0 Hz (marked as E), and decreasing to 0 Hz (marked as F). For the first four conditions, the RF variation range of each data is set as small, medium, and large. Two different maximum RF values, i.e., 5 Hz and 10 Hz, are set for the last two conditions. Meanwhile, for data under the same RF variation mode and range, the RF variation speed is introduced in three different trials to explore the influence of angular acceleration on FCFBI. According the above experimental settings, a total of forty-eight data sets are collected. All data are sampled at 8000 Hz. The details of the bearing outer race fault data set can be found in Table A2.

**Figure 17:**
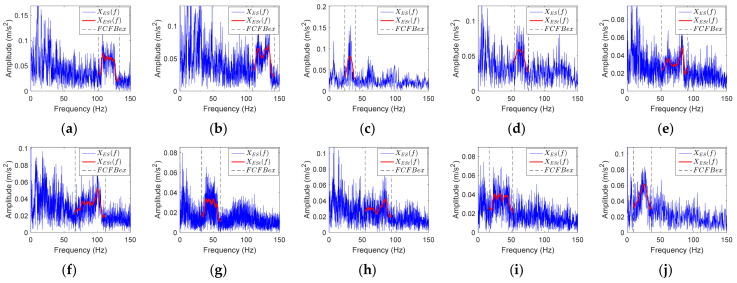

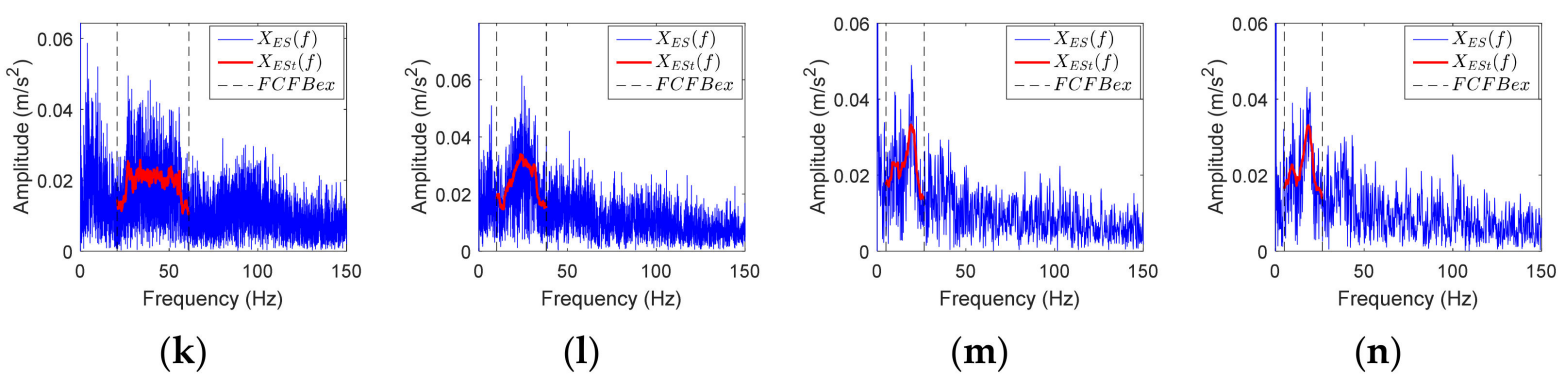
Envelope spectra of the outer race fault vibration signals. (**a**) S-A-2; (**b**) S-B-2; (**c**) S-C-2; (**d**) S-D-2; (**e**) M-A-2; (**f**) M-B-2; (**g**) M-C-2; (**h**) M-D-2; (**i**) L-A-2; (**j**) L-B-2; (**k**) L-C-2; (**l**) L-D-2; (**m**) E-2; (**n**) F-2.

Since the vibration transmission path from bearing 2 to the base is complicated, the SNR of the vibration signal collected by sensor 5 is lower [11]. For this reason, the vibration signal collected by sensor 5 is selected in this experiment to validate the performance of FCFBI under low SNR. A preliminary analysis of the envelope spectrum is performed on all signals. Fourteen data are taken as examples, the corresponding envelope spectra in the low frequency band are exhibited in Figure 17. Due to the stronger noise interference in the base sensor signals, FCFB is less distinct than that in the inner race fault experiment. It should be noted that under conditions E and F, RFB appears in the envelope spectrum in [0, max(*f_r_*)] due to shaft misalignment, which causes the 1st FCFB and RFB to overlap. Meanwhile, DC component interference also exists in the envelope spectrum. Hence, [max(*f_r_*), *FCC*·max(*f_r_*) + *ex*] is defined as the *FCFBex* for these two conditions. The red curves in Figure 17 display the trend of each envelope spectrum in *FCFBex* when setting *wl* = 30. The fitted curves reveal the variation trend of the envelope spectrum in *FCFBex* and weaken the amplitude oscillation. Similarly, the trends of the fault templates are fitted with *mdp* = 20.

**Figure 18:**
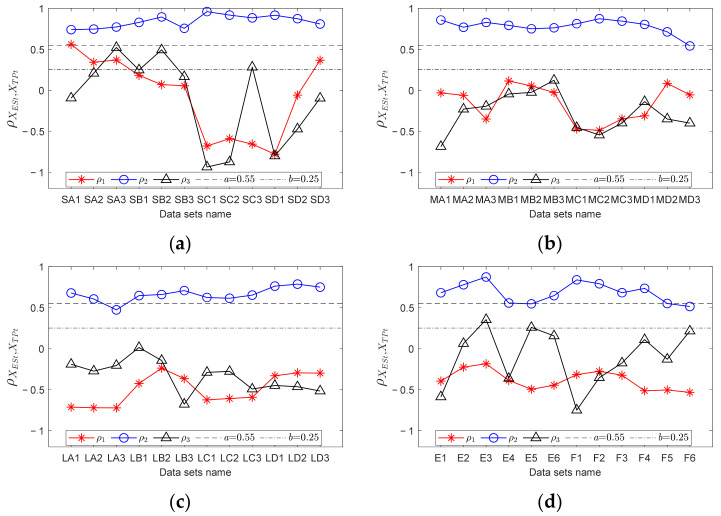
Diagnostic results of outer race fault data with trend fitting. (**a**) Small RF variation range data set; (**b**) Medium RF variation range data set; (**c**) Large RF variation range data set; (**d**) Data sets with RF increasing from 0 Hz and decreasing to 0 Hz.

For each data, calculate the correlation coefficients between the trend curves of the envelope spectrum fault templates in the corresponding *FCFBex*. The upper and lower limit of the correlation coefficient are set the same as that in experiment one. Figure 18a shows the diagnostic results of small RF range data. Among the twelve data sets, six data sets, i.e., S-B-1, S-B-3, S-C-1, S-C-2, S-D-1 and S-D-2, can be diagnosed as outer race faults. Although the correlation coefficients between the other six data sets’ envelope spectra and outer race templates are higher than the given upper limit, the correlation coefficients between the envelope spectra and other fault templates are higher than the lower limit; therefore, these data cannot be determined as outer race faults. The diagnostic results of medium RF range data are illustrated in Figure 18b. Except for M-D-3, other eleven data sets can be identified as outer race faults. Figure 18c depicts the results of large RF range data sets. All data except L-A-3 can be recognized as outer race faults. The diagnostic results of data sets under conditions E and F depicted in Figure 18d suggest that except for E-3, E-5, and F-6, the nine other data sets are successfully diagnosed.

**Figure 19:**
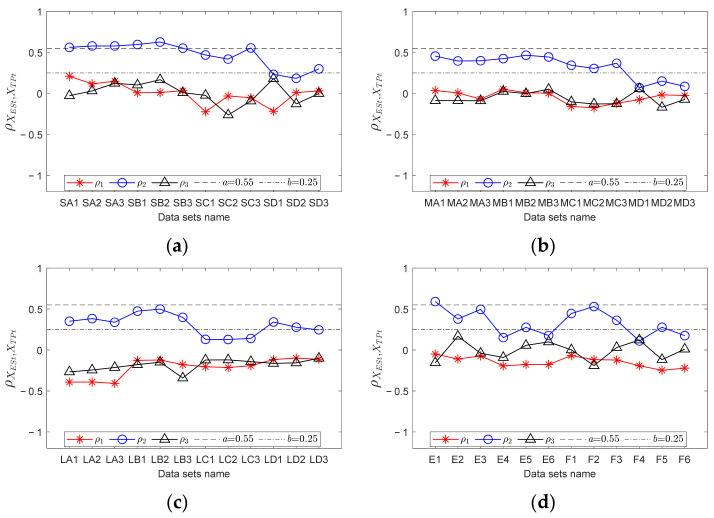
Diagnostic results of outer race fault data without trend fitting. (**a**) Small RF variation range data set; (**b**) Medium RF variation range data set; (**c**) Large RF variation range data set; (**d**) Data sets with RF increasing from 0 Hz and decreasing to 0 Hz.

Similarly, the correlation coefficients between the original envelope spectra and original fault templates in *FCFBex* are also calculated for comparison. The results of the small RF variation range data shown in Figure 19a indicate that data under conditions A and B together with S-C-3 can be diagnosed as outer race faults. Figure 19b,c suggest that no data under the medium and large RF variation ranges can be identified correctly. From the diagnostic results under conditions E and F depicted in Figure 19d, only E-1 is correct.

To sum up, in the bearing outer race fault experiment, the diagnostic accuracies of FCFBI and FCFBI without trend fitting are 77.1% (37 out of 48 data) and 14.6% (7 out of 48 data), respectively. Their corresponding average correlation coefficients are 0.741 and 0.359, respectively. Therefore, for bearing outer race faults under VS, the above comparative analysis verifies that FCFB feature enhancement has a positive effect on improving the diagnostic accuracy based on FCFBI.

## 5. Comparison Verification and Discussion

In order to further validate the superiority of the proposed FCFBI method, this section introduces two representative methods from OT and TFA, namely, COT [12] and MTFCE [22], for comparison verification. The performance of FCFBI and these two comparison methods is also discussed and summarized.

### 5.1. Comparison Method One: COT

The idea of COT is to re-sample the signal in time domain at equal angle intervals with reference to the speed signal to transform the non-stationary signals from the time domain to the order domain, where the signal exhibits a pseudo-stationary property. COT focuses on solving the resampling time through the speed signal. Assuming that the angular acceleration of the shaft is constant, the shaft rotational angle *θ* can be expressed by the quadratic equation of time *t*:

(13) $$\theta (t)=b_{0}+b_{1}t+b_{2}t^{2}$$

where *b*_0_, *b*_1_, and *b*_2_ are three unknown coefficients, which can be solved by the following equation in matrix form:

(14) $$\left(\begin{array}{c} 0\\ \Delta \Phi \\ 2\Delta \Phi \end{array}\right)=\left[\begin{array}{lll} 1 & t_{1} & t_{1}^{2}\\ 1 & t_{2} & t_{2}^{2}\\ 1 & t_{3} & t_{3}^{2} \end{array}\right]\left\{\begin{array}{l} b_{0}\\ b_{1}\\ b_{2} \end{array}\right\}$$

In Equation (14), *t*_1_, *t*_2_, and *t*_3_ are the keyphasor arrival times corresponding to three consecutive equiangular increments ΔΦ, which can be figured out from the speed signal. After obtaining *t*_1_, *t*_2_, and *t*_3_, calculate the value of the equiangular increment Δ*θ* for resampling, insert the values into Equation (13), and then the time of the *m*th (*m* = 1, 2,…, *k*) resampling point can be solved:

(15) $$t_{m}=\frac{1}{2b_{2}}\left[\sqrt{4b_{2}\left(m\Delta \theta -b_{0}\right)+b_{1}^{2}}-b_{1}\right]$$

After all resampling times are obtained, the time domain signal can be transformed to the angle domain by interpolation at these time points. In this paper, let ΔΦ = 2*π*, *k* = 1024; the cubic spline is chosen for interpolation. Finally, envelope spectrum analysis is performed on the angle domain signal. Fault diagnosis can be accomplished by observing the order envelope spectrum peak amplitude near FCO and its harmonic orders.

**Figure 20:**
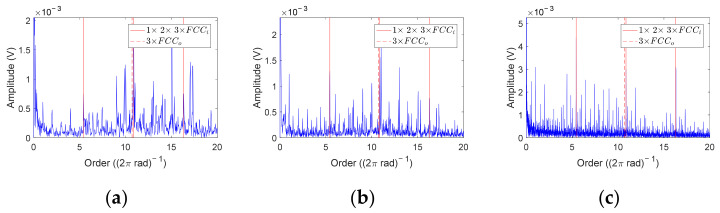
Order envelope spectra of inner race fault data. (**a**) S-A-1; (**b**) M-A-1; (**c**) L-A-1.

**Figure 21:**
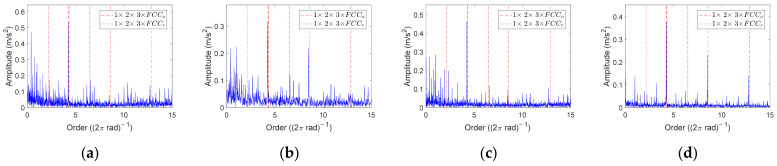
Order envelope spectra of outer race fault data. (**a**) S-A-1; (**b**) S-D-1; (**c**) M-B-1; (**d**) L-D-2.

Firstly, COT is performed on the inner race fault data sets. Figure 20 shows three representative order envelope spectra selected from twenty-eight data sets. The red solid line represents *FCC_i_* and its harmonic orders, and the red dashed line represents 3 × *FCC_o_*. It can be found that 2 × *FCC_i_* = 10.86, which is very close to 3 × *FCC_o_* = 10.71. If the amplitude at *FCC_i_* and 3 × *FCC_i_* is not significant enough or other disturbances are present, the data cannot be diagnosed as single inner race faults, such as the order envelope spectrum of S-A-1 shown in Figure 20a. For other data, such as the order envelope spectra of M-A-1 and L-A-1 shown in Figure 21b,c, there are marked peaks at *FCC_i_* and 3 × *FCC_i_*, while no obvious peaks appear at *FCC_o_* and 2 × *FCC_o_*. Therefore, these data can be diagnosed as inner race faults.

Then, COT is employed to analyze the outer race fault data sets. It is worth noting that 2 × *FCC_r_* and *FCC_o_* of the NU204 bearing are very close; their values are 4.32 and 4.29, respectively. For the bearing vibration signal under constant speed, if the rolling element has defects, the peak amplitude in the envelope spectrum will be more obvious at 2 × *FCC_r_*·*f_r_*(*t*) [38]. For the same reason, the peak amplitude in the order envelope spectrum will be more obvious at 2 × *FCC_r_* when the rolling element fault occurs. Therefore, in the outer race fault experiment, if there is a peak around 4.3 in the order envelope spectrum, it is difficult to judge whether the bearing has outer race faults, rolling element faults or compound faults. It is necessary to combine the amplitudes at harmonic orders of *FCC_r_* and *FCC_o_* for further judgment. Through the analysis of order envelope spectra, we can find that an obvious peak amplitude around 4.3 appears in all forty-eight data sets. Therefore, according to the amplitude at 3 × *FCC_r_*, 2 × *FCC_o_*, and 3 × *FCC_o_*, the order envelope spectra of all data can be classified into four types, and their representatives are illustrated in Figure 21. The details of these order envelope spectra and the corresponding diagnostic results are listed in Table 4. Y indicates that there is an obvious peak at the corresponding FCC, while N indicates a weak peak or no peak. In total, thirty out of forty-eight data sets can be diagnosed as outer race faults.

### 5.2. Comparison Method Two: MTFCE

Based on TFA and ridge extraction, MTFCE extracts ISRF and IFCF ridges from TFR of bearing vibration signal under VS and identifies faults by the average ratios of the IFCF curves and ISRF curve. MTFCE includes two main steps. In the first step, multiple time-frequency curves are extracted from the STFT-based TFR of the vibration envelope signal using fast path optimization ridge extraction [39]. The curve with the lowest frequency is treated as ISRF. Then, we calculate the average ratios of the point-to-point frequency of each extracted curve to ISRF and compare these ratios with theoretical FCC, limiting the relative error *er* between average ratios and FCC and the variance *var* of point-to-point frequency ratios to realize preliminary fault diagnosis. Proceed to the second step if the fault cannot be detected in the first step, that is, replace ISRF in first step by the curve with the lowest frequency in the original vibration, TFR, and repeat the first step for further fault detection. If the fault is still not identified, the bearing is considered healthy. The MATLAB code of MTFCE used in this section is from [22].

Firstly, MTFCE is performed on the inner race fault data sets. Refer to the parameter settings in [22]: set the number of ridges *m* = 4, STFT window length *w* = 7000, overlap *ol* = 6800 for S-C-1 and S-C-2; set *w* = 9000, *ol* = 8000 for S-C-3; set *w* = 9000, *ol* = 8800 for other data. For all data, set the relative error between the average ratios and *FCC_i_* as *er*_*i* = 0.02, with the corresponding variance as *var*_*i* = 0.11; set the relative error between average ratios and *FCC_o_* as *er*_*o* = 0.055 and the corresponding variance as *var*_*o* = 0.09. The diagnostic results of all data sets are listed in Table 5.

**Figure 22:**
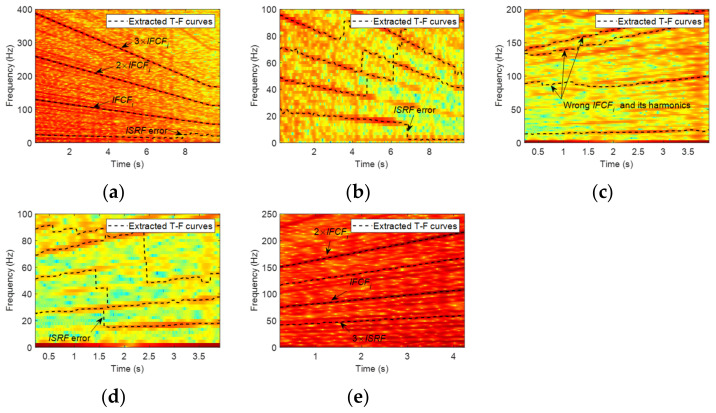
TFRs and the extracted MTFCs of inner race fault data. Envelope signal TFR of (**a**) L-B-1; (**c**) M-A-1; (**e**) M-A-3. Original signal TFR of (**b**) L-B-1; (**d**) M-A-1.

From the results in Table 5, it can be seen that 15 of the 28 data sets are successfully diagnosed as inner ring faults. Eleven data sets are diagnosed as healthy. Among these 11 data sets, there are 9 data sets where IFCF and its harmonic ridges can be identified successfully in the envelope TFR (see Figure 22a for an example), while ISRF is not identified or has large errors in both the envelope TFR and original TFR (Figure 22b), causing wrong results. For two other data sets, the identified IFCF, its harmonics, and ISRF have large errors in the envelope TFR (Figure 22c). At the same time, the ISRF ridges cannot be successfully extracted from the original TFR (Figure 22d), resulting in misdiagnosis. There are two data sets that are wrongly diagnosed as outer race faults. The reason is that 3 × *ISRF* curves are extracted from the envelope TFR with the lowest frequency, and *IFCF_i_* and 2 × *IFCF_i_* are also derived at the same time; because (2 × *IFCF_i_*/3 × *ISRF*) ≈ (3 × *IFCF_o_*/3 × *ISRF*) = *FCC_o_*, they are misdiagnosed as outer race faults (Figure 22e).

Then, MTFEC is utilized to analyze the outer race fault data sets. The number of ridges are set as *m* = 4. For vibration signals with duration less than 5 s, set *w* = 5000, *ol* = 4900; set *w* = 9000, *ol* = 8900 for other. For all data, set *er*_*i* = 0.02, *var*_*i* = 0.11, *er*_*o* = 0.06, *var*_*o* = 0.1. Moreover, we introduced the judgment of the rolling element fault based on the MATLAB code provided in [22]. We set the error and variance corresponding to rolling element faults as *er*_*r* = 0.06, *var*_*r* = 0.1. The diagnostic results of 48 data sets are listed in Table 6.

**Figure 23:**
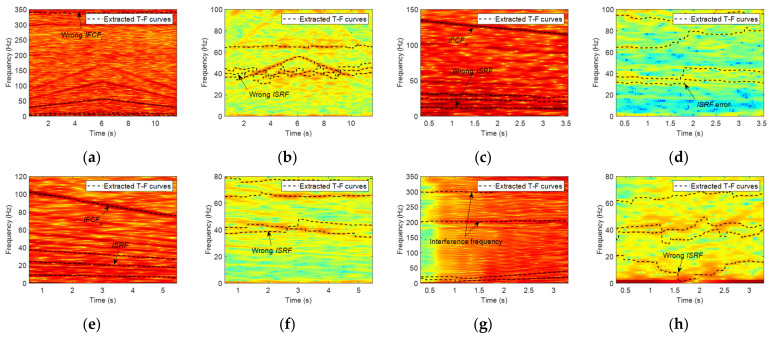
TFRs and the extracted MTFCs of outer race fault data. Envelope signal TFR of (**a**) L-C-1; (**c**) S-B-1; (**e**) M-B-1; (**g**) E-1. Original signal TFR of (**b**) L-C-1; (**d**) S-B-1; (**f**) M-B-1; (**h**) E-1.

The diagnosis results in Table 6 show that only four data sets can be diagnosed as outer race faults. Three data sets are identified as outer race faults by coincidence. After analyzing the process, it was found that the IFCF and ISRF extracted in TFR are incorrect (see Figure 23a,b for an example). On the contrary, the ratio and the variance of the wrong curves only satisfy the preset *er*_*o* and *var*_*o*, leading to misdiagnosis, so these three data sets should not be classified as correct. Three data sets are misidentified as rolling element faults for a similar reason, i.e., ‘Outer race fault by coincidence’. Thirty-eight data sets are incorrectly identified as healthy. Among them, there are 21 data sets in which IFCF ridges can be extracted successfully in the envelope TFR, but the ISRF extracted in both the envelope and original TFR is incorrect or inaccurate (Figure 23c,d). Although there are seven data sets in which IFCF and ISRF can be identified in the envelope TFR (Figure 23e), ridges with the lowest average frequency are not the true ISRF or TFR (Figure 23e,f). In the remaining 10 data sets, RF under condition E or F is affected by noise at around 200 Hz and 300 Hz in the envelope TFR (Figure 23g), leading to the misidentification of IFCF. At the same time, ISRF cannot be extracted from TFR due to relatively low RF and poor frequency resolution (Figure 23h).

### 5.3. Discussion

Based on the experimental results of FCFBI and two comparison methods, the discussion is carried out from five aspects: theoretical basis, diagnostic accuracy, diagnostic time, tachometer dependence, and the automatic degree. The discussion results are summarized in Table 7.

On a theoretical basis, COT is based on the pseudo-stationary property of the vibration signal in the angular domain, and completes domain transformation by resampling. However, it is affected by error and close order. MTFCE is based on the TFA of non-stationary signals and the time-frequency ridge extraction algorithms. Nevertheless, TFA algorithms are inevitably limited by the time-frequency resolution. Ridge extraction algorithms are susceptible to noise interference, which makes it difficult to extract ISRF located in the low frequency band of TFR. Besides, the fault diagnosis strategy based on the average ratio of point-to-point frequency is also easy to misdiagnose. The proposed FCFBI is based on the envelope FCFB identification. First of all, the simulation signal and formula derivation verify that FCFB exists in the envelope spectrum of the bearing fault vibration signal under VS, and it is related to the fault type and RF. Secondly, the correlation coefficients between the envelope spectrum and different fault templates are calculated only in the 1st extended FCFB, which can not only reveal the possibility of different fault types but can also effectively avoid the misdiagnosis problem in COT and MTFCE.

In the aspect of diagnostic accuracy, COT achieves high accuracy for inner race fault data sets, while the accuracy for outer race fault data sets is significantly reduced. The main reason is that *FCC*_o_ and 2 × *FCC_r_* of the NU204 bearing are very close. Moreover, the SNR of the vibration signal collected from the base is low, which will also reduce the accuracy of COT. MTFCE has the lowest accuracy among the three methods, which is mainly due to the limitation of the time-frequency resolution of the TFA algorithm and the inaccuracy of ridge extraction caused by noise. FCFBI achieves relatively high diagnostic accuracy for both inner race and outer race fault data sets. Compared with COT, although the diagnostic accuracy for inner race fault data sets is lower, the accuracy of outer race fault data sets is higher. This shows that FCFBI can obtain relatively high and more stable diagnostic accuracy in spite of differences in bearing types, fault types, and signal SNRs.

Regarding the evaluation of the diagnostic time, all three methods are implemented in MATLAB R2016b. The configuration of the computer is an Intel^®^ Core™ i7_7700HQ CPU @ 2.80 GHz, 8G RAM. The diagnostic time of COT cannot be measured in seconds because it requires manual observation of the spectra. MTFCE takes the shortest time to process inner race fault data sets, while it takes the longest time to process outer race fault data sets. The total diagnostic time of MTFCE is the longest. Although the diagnostic time of FCFBI is longer than MTFCE for inner race fault data sets, it takes the shortest time for outer race fault data sets, and the total diagnostic time is the shortest.

Turning to the discussion of tachometer dependence, it is clear that MTFCE does not require a tachometer, but the accuracy of the estimated ISRF is affected by the TFA and ridge extraction algorithms. Although COT and FCFBI require a tachometer to provide speed signals, their reliance on the tachometer is different. COT is more dependent on the tachometer because tiny speed errors will affect the accuracy of resampling time, which in turn affects the accuracy of the order envelope spectrum. FCFBI is less dependent on the tachometer than COT owing to the FCFB in the envelope spectrum and fault templates that are mainly affected by the RF variation range. When the minimum and maximum RF values are correct, the range of FCFB in the envelope spectrum and the fault templates will be correct, while RF fluctuation will only influence the amplitude inside FCFB. After trend fitting, the influence of RF fluctuation will be eliminated to a large extent. Moreover, extending the FCFB also increases the tolerance to RF errors. In addition, for some specific mechanical equipment, if the speed variation is stable and the working RF range is known in advance, fault diagnosis can be completed through the estimated or hypothetical RF curve even without a tachometer.

The main factors affecting the automatic degree of the above three methods are the number of preset parameters and whether diagnostic results can be automatically output. For COT, there are two parameters that need to be preset. Their value will affect the order envelope spectrum resolution, which is very important for the accuracy of the identification of corresponding spectral lines. In addition, COT implements fault diagnosis by manually observing the order envelope spectrum, which is not automatic. Although MTFCE can automatically output diagnostic results, the number of preset parameters reaches nine. The first three parameters in Table 7 affect the time-frequency resolution of TFR and the number of ridges. The last six parameters influence the diagnostic results, and there are no clear rules to follow for setting these parameters for different types of mechanical equipment. Therefore, the automatic degree of MTFCE is moderate. The proposed FCFBI can also automatically output diagnostic results. There are basic rules to follow for the five preset parameters. In general, the values of *wl* and *mdp* should increase with the data length, and the value of *ex* can increase with the FCFB interval range. For *a* and *b*, the appropriate value can be determined only with trial and error. It is worth noting that for each experimental data sets in chapter 4, although all data lengths are different, the values of *wl*, *mdp,* and *ex* remain the same for each data set. The values of *a* and *b* are the same for all data sets. This indicates that FCFBI is less affected by the preset parameters and has a high degree of automation.

## 6. Conclusions

In this paper, a new fault diagnosis method for rolling element bearing under variable speed is proposed based on envelope spectrum fault characteristic frequency band identification. We reveal the envelope spectrum characteristics of bearing vibration signals under variable speed. Furthermore, the correlation coefficient-based FCFBI and FCFB feature enhancement enables bearing fault diagnosis to be directly performed through envelope spectrum, which provides a new idea and simplifies the bearing fault diagnosis process under VS.

The proposed FCFBI method is preliminarily verified by bearing fault simulation signals under VS, and 7 out 10 signals can be successfully diagnosed even under very low SNRs. Two experiments are implemented on different test benches, which provide 28 inner race fault and 48 outer race fault experimental data sets that cover the complex working conditions. Their diagnostic accuracy is 78.6% and 77.1%, respectively. The experimental results suggest that FCFBI can achieve relatively high fault diagnostic accuracy despite the differences in bearing types and fault types. In addition, by comparing with COT and MTFCE, it is further verified that FCFBI overcomes the shortcomings of traditional methods to some extent. Good performance balance of FCFBI in terms of diagnostic accuracy, calculation time, tachometer dependence, and automatic degree is also confirmed.

However, the method proposed in this paper still has some weaknesses and limitations. For the case of bearing vibration under a small rotational frequency variation range, the diagnostic accuracy of FCFBI needs to be improved. FCFBI does not perform noise reduction and preprocessing on the original signal, and the combination with advanced noise reduction methods will be studied in future to improve the detection rate. All experiments were carried out on the test bench, and industrial field data need to be employed to validate the proposed method in future research.

## Figures and Tables

**Table 1:** Parameters used for bearing simulation signals under VS.

*α*	*A_0_*	*η*	*β*	*ω_r_*	*T_t_*	SNR	*FCC_i_*	*FCC_o_*	*FCC_r_*
0.3	1	0.05	1500	5000 Hz	3 s	−5 dB	5.5	3.5	2.3

**Table 2:** Formula of different *f_r_*(*t*) values.

RF Variation Modes	Formula
Linear increasing	$f_{r1}(t)=4t+35$
Linear decreasing	$f_{r2}(t)=-4t+47$
Non-linear increasing	$f_{r3}(t)=-4t^{2}/3+8t+35$
Non-linear decreasing	$f_{r4}(t)=4t^{2}/3-8t+47$
Non-linear increasing then decreasing	$f_{r5}(t)=-16t^{2}/3+16t+35$
Non-linear decreasing then increasing	$f_{r6}(t)=16t^{2}/3-16t+47$
**RF ranges**	**Formula**
Large variation range	$f_{r7}(t)=8t+15$
Small variation range	$f_{r8}(t)=t+35$
Increasing from 0 Hz	$f_{r9}(t)=4t$
Decreasing to 0 Hz	$f_{r10}(t)=-4t+12$

**Table 3:** Geometric parameters and FCF of the NU204 bearing.

*D*/mm	*d*/mm	*z*	*ϕ*/°	*FCC_i_*	*FCC_o_*	*FCC_r_*
34	7.5	11	0	6.71	4.29	2.16

**Table 4:** Diagnostic results of outer race fault data sets based on COT.

Type	Peak Amplitude at			Data Sets	Diagnostic Results
	2 × *FCC_o_*	3 × *FCC_o_*	3 × *FCC_r_*		
1	N	N	Y	S-A-1, S-A-2, S-A-3, S-B-1, S-B-2, S-B-3	Outer race, rolling element or compound faults (6 data)
2	Y	N	Y	S-D-1, S-D-2, S-D-3, M-A-1, M-A-3, M-D-1, M-D-2, M-D-3	Outer race, rolling element or compound faults (8 data)
3	Y	Y	Y	M-A-2, M-B-1, M-B-2, M-B-3	Outer race, rolling element or compound faults (4 data)
4	Y	Y	N	S-C-1, S-C-2, S-C-3, M-C-1, M-C-2, M-C-3, all data under L, E, and F	Outer race fault (30 data)

**Table 5:** Diagnostic results of inner race fault data sets based on MTFEC.

Diagnostic Results	Data Sets
Inner race fault (15 data sets)	S-A-1, S-A-3, S-B-2, S-C-1, S-C-2, S-C-3, M-A-2, M-C-1, M-C-2, M-C-3, M-D-1, M-D-2, M-D-3, L-A-3, L-B-3
Healthy (11 data sets)	S-A-2, S-B-1, S-B-3, S-D-1, S-D-2, S-D-3, M-A-1, M-B-2, M-B-3, L-A-1, L-B-1
Outer race fault (2 data sets)	M-A-3, M-B-1

**Table 6:** Diagnostic results of outer race fault data sets based on MTFEC.

Diagnostic Results	Data Sets
Outer race fault (4 data)	S-B-2, S-C-1, L-A-2, L-A-3
Outer race fault by coincidence (3 data sets)	L-C-1, F-1, F-4
Rolling element fault (3 data sets)	S-A-1, M-D-1, M-D-2
Healthy (38 data sets)	Others

**Table 7:** Discussion results of FCFBI and two comparison methods.

Method	Theoretical Basis	Diagnostic Accuracy (Inner|Outer Race Fault, %)	Diagnostic Time (Inner|Outer Race Fault|Total Time, s)	Tachometer Dependence	Automatic Degree	
					Preset Parameters	Automatic Diagnosis
COT	Pseudo-stationary vibration signal in angular domain	96.4|62.5	-	High	ΔΦ, *k*	No
MTFCE	TFA of the non-stationary signal and ridge extraction	53.6|8.3	85.6|218.5|304.1	No	*m*, *w*, *ol*, *er_i*, *er_o*, *er_r*, *var_i*, *var_o*, *var_r*	Yes
FCFBI	FCFB in the envelope spectrum	78.6|77.1	187.8|87.2|275	Low	*wl*, *mdp*, *ex*, *a*, *b*	Yes

## Data Availability

The bearing data from the University of Ottawa can be found in [37]. The bearing data from Beijing Jiaotong University are available on request from the corresponding author.

## Appendix A

Table A1 lists the names and RF ranges of bearing inner race fault data in Experiment one after re-segmentation of the original data from the University of Ottawa. The naming rule of data follows ‘RF variation range-RF variation mode-number of trials’, where the RF variation range marker S, M, and L represent small, medium, and large, respectively. The RF variation mode markers A, B, C, and D represent increasing, decreasing, increasing then decreasing, and decreasing then increasing, respectively. The content in parentheses after the data name indicates the specific RF variation in Hertz.

**Table A1:** Details of bearing inner race fault data sets.

RF Range	RF Variation Mode			
	Increasing	Decreasing	Increasing then Decreasing	Decreasing then Increasing
Small	S-A-1 (12.5–15.0) S-A-2 (12.9–15.5) S-A-3 (13.4–16.2)	S-B-1 (12.1–10.1) S-B-2 (15.8–13.2) S-B-3 (14.4–12.0)	S-C-1 (20.0–24.0–20.0) S-C-2 (19.3–26.1–19.3) S-C-3 (17.8–21.4–17.8)	S-D-1 (18.8–15.2–18.2) S-D-2 (18.4–15.4–18.4) S-D-3 (19.4–16.2–19.4)
Medium	M-A-1 (12.5–18.7) M-A-2 (12.9–19.4) M-A-3 (13.5–20.2)	M-B-1 (15.2–10.1) M-B-2 (19.7–13.2) M-B-3 (18.0–12.0)	M-C-1 (16–24–18.5) M-C-2 (15.4–23.1–17.9) M-C-3 (14.8–21.4–14.3)	M-D-1 (22.8–15.2–19.4) M-D-2 (23.1–15.4–19.8) M-D-3 (23.0–16.2–23.6)
Large	L-A-1 (12.5–27.8) L-A-3 (13.5–28.5)	L-B-1 (24.3–9.9) L-B-3 (25.8–12.0)	-	-

Table A2 lists the names and RF ranges of bearing outer race fault data in Experiment two. The duration of each experimental signal is listed in parentheses in seconds, and the RF variation is also listed in Hertz.

**Table A2:** Details of bearing outer race fault data sets.

RF Range	RF Variation Mode			
	Increasing	Decreasing	Increasing then Decreasing	Decreasing then Increasing
Small	25.0–30.0 Hz S-A-1 (3.60 s) S-A-2 (4.80 s) S-A-3 (6.01 s)	32.0–26.7 Hz S-B-1 (3.86 s) S-B-2 (5.14 s) S-B-3 (6.44 s)	6.7–8.00–6.7 Hz S-C-1 (2.20 s) S-C-2 (3.00 s) S-C-3 (4.00 s)	S-D-1 (16.8–14.0–16.8 Hz, 2.76 s) S-D-2 (16.6–13.8–16.6 Hz, 3.43 s) S-D-3 (16.5–13.7–16.5 Hz, 4.38 s)
Medium	13.3–20.0 Hz M-A-1 (4.80 s) M-A-2 (6.41 s) M-A-3 (8.01 s)	25.0–16.7 Hz M-B-1 (6.01 s) M-B-2 (8.02 s) M-B-3 (10.02 s)	8.8–13.2–8.8 Hz M-C-1 (7.54 s) M-C-2 (10.31 s) M-C-3 (13.77 s)	M-D-1 (21.1–14.0–21.1 Hz, 6.06 s) M-D-2 (20.8–13.8–20.8 Hz, 7.88 s) M-D-3 (20.6–13.7–20.6 Hz, 10.26 s)
Large	5.0–11.0 Hz L-A-1 (4.32 s) L-A-2 (5.76 s) L-A-3 (12.00 s)	7.3–3.3 Hz L-B-1 (2.88 s) L-B-2 (3.84 s) L-B-3 (4.80 s)	6.0–13.2–6.0 Hz L-C-1 (12.15 s) L-C-2 (16.60 s) L-C-3 (22.14 s)	L-D-1 (7.9–3.6–7.9 Hz, 10.08 s) L-D-2 (7.7–3.5–7.7 Hz, 13.11 s) L-D-3 (7.8–3.6–7.8 Hz, 17.84 s)
Increasing from 0 Hz	0–5 Hz E-1 (3.60 s) E-2 (4.82 s) E-3 (6.01 s) 0–10 Hz E-4 (7.20 s) E-5 (9.62 s) E-6 (12.01 s)			
Decreasing to 0 Hz	5–0 Hz F-1 (3.60 s) F-2 (4.81 s) F-3 (6.01 s) 10–0 Hz F-4 (7.20 s) F-5 (9.61 s) F-6 (12.02 s)			
